# Total Hip and Knee arthroplasty in a patient with osteopetrosis: a case report and review of the literature

**DOI:** 10.1186/s12891-015-0716-x

**Published:** 2015-09-21

**Authors:** Lin Xie, Fan Ding, Jing Jiao, Wusheng Kan, Junwen Wang

**Affiliations:** Department of Orthopedic Surgery, Wuhan Orthopedic Hospital, Wuhan Puai Hospital, Huazhong University of Science and Technology, Hanzheng Street 473#, Wuhan, 430033 Hubei Province China

**Keywords:** Total hip arthroplasty (THA), Total knee arthroplasty (TKA), Osteopetrosis

## Abstract

**Background:**

Osteopetrosis is an uncommon, inherited disease, characterized by osteosclerosis, obliteration of the medullary canal, calcified cartilage and brittle bone due to impaired osteoclast resorption. Osteoarthritis is common in patients with osteopetrosis. If the patient has pain and dysfunction, total joint arthroplasty is often the treatment of choice but presents many intraoperative and postoperative challenges. Few studies have presented both Total hip arthroplasty (THA) and Total knee arthroplasty (TKA) in one patient. This article describes a case of left hip osteoarthritis and right knee osteoarthritis in a patient with osteopetrosis. We performed THA and TKA in a 59-year-old osteopetrotic patient with painful osteoarthritis in the left hip and right knee.

**Case presentation:**

A 59-years-old female with osteopetrosis was referred to our department because of a history of left hip pain and bilateral, right greater-than-left, knee pain with activity limitation for 13 years. She had no fracture history. In our hospital the patient underwent THA in the left hip firstly. Six months later, we performed TKA of the right knee. At 15-months follow-up, the components were in good position, and the patient could walk freely and perform activities of daily living with no pain.

**Conclusions:**

This case report demonstrates that total joint arthroplasty is an effective treatment for painful hip and knee osteoarthritis in patients with osteopetrosis.

## Background

Osteopetrosis is an uncommon, inherited disease originally described in 1904 by Albert Schönberg, a German radiologist [1]. In most forms, it is characterized by osteosclerosis, obliteration of the medullary cavity, calcified cartilage and brittle bone due to impaired osteoclast function.

Osteopetrosis has been classified into three main types: infantile malignant, intermediate and benign. The malignant [2, 3] autosomal recessive form of this disease results in death in the first decade of life because of obliteration of the medullary cavity, which results in loss of bone marrow, pancytopenia, immunodeficiency and life-threatening infections [4, 5]. The intermediate autosomal recessive form carries a life expectancy into adulthood. This type has the highest incidence of osteomyelitis of the jaw attributed to the decreased bone vascularity and the relative low white blood cells. The benign autosomal dominant form is the most common. It typically carries a full life expectancy, despite of increased propensity for fractures and other musculoskeletal problems such as hip and knee osteoarthritis.

THA and TKA remains a good option for treating painful osteoarthritis in osteopetrotic patients. However, there were several technical challenges associated with the surgery, including sizing of the component, preparation of the abnormal medullary canal, press-fit fixation of all the components and inadequate surfaces for cement interdigitation. We had to take precautions in order to avoid iatrogenic fracture, overheating and breakage of drills and saws. Postoperatively there was an increased risk for osteomyelitis attributed to the lack of marrow vascularity and impairment of white cell function and longer surgical time.

To date, few studies have presented arthroplasty in in patients with osteopetrosis. This article is, to our knowledge, the first description of both THA and TKA in a patient with osteopetrosis. After more than a year follow up time, patient showed excellent clinical function and remained satisfied with the surgical outcome.

## Case presentation

**Fig. 1:**
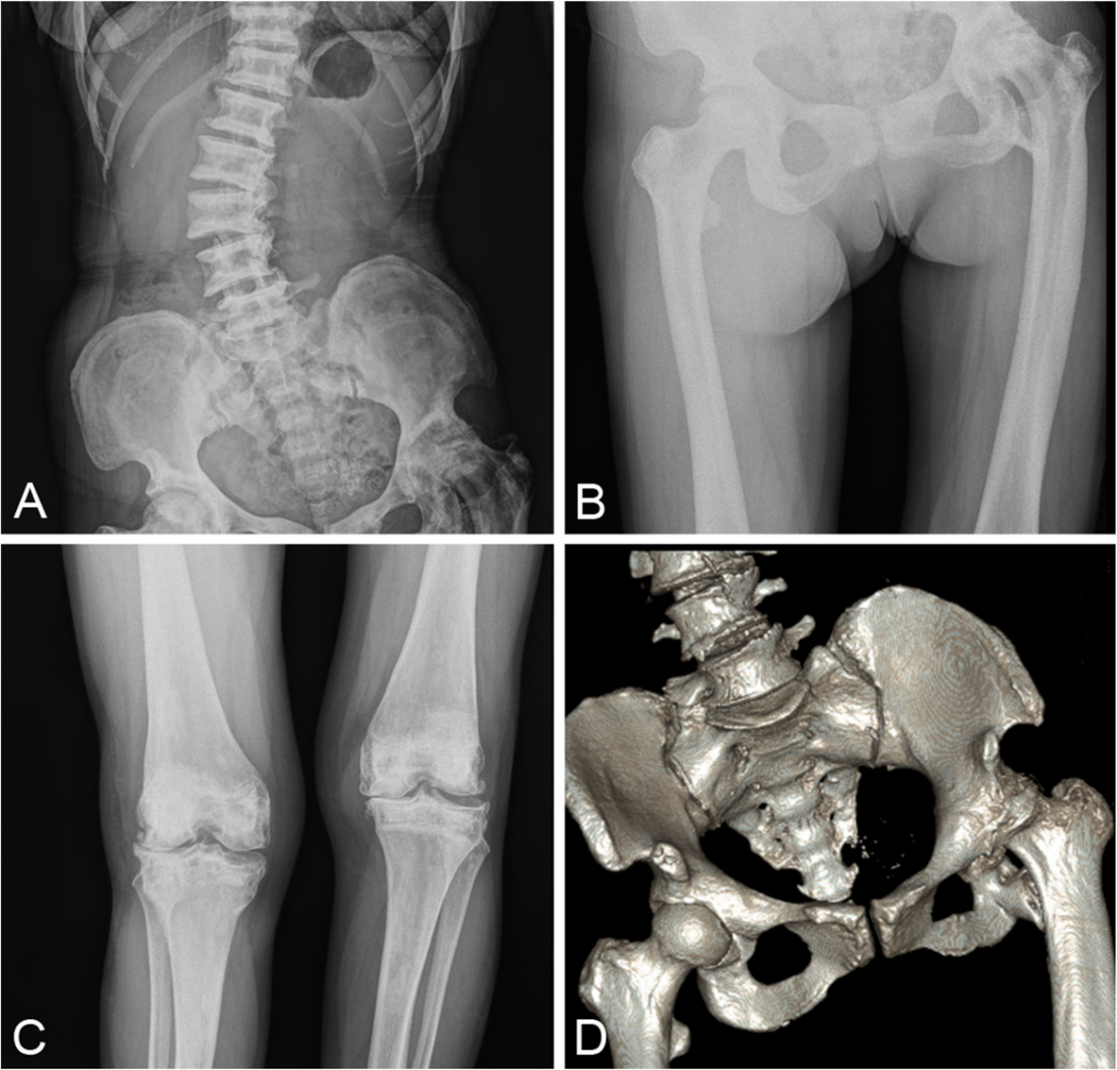
Preoperative radiographs of the spine, pelvic and knee. **a, b, d** X ray of pelvic showed endobones, and x ray of spine showed “rugger jersey spine”. The x-rays also showed severe osteoarthritis in the left hip with significant acetabular erosion, proximal migration of the femoral head and reduced joint space. **c** X-rays of the right knee showed similar osteoarthritic changed

**Fig. 2:**
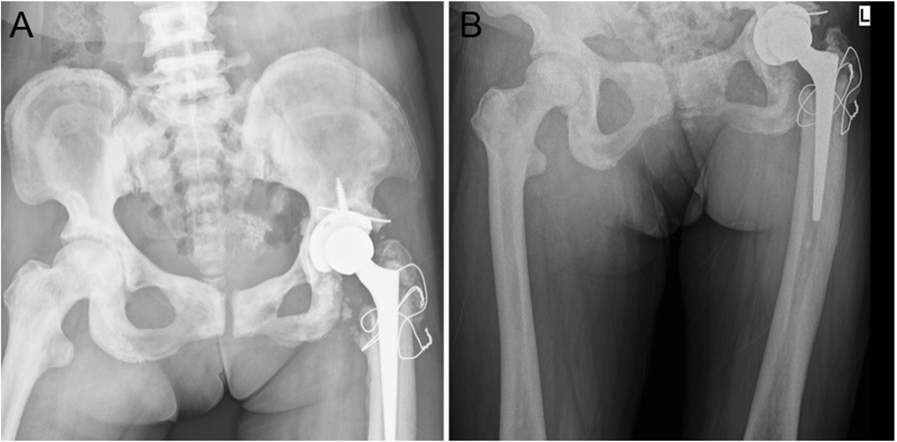
Postoperative radiograph of the left hip. The greater trochanter accidentally broke off and was fixed with steel-wire

**Fig. 3:**
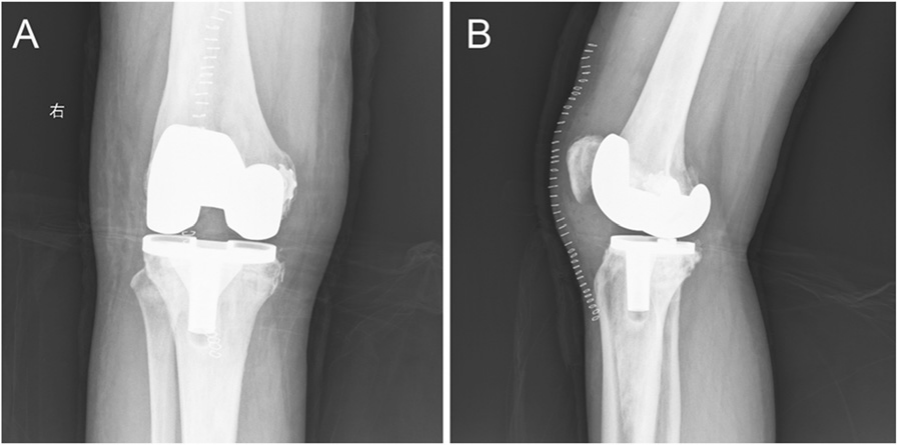
Postoperative radiograph of the right knee. An avulsion fracture occurred on the femoral medial epicondyle. A small spider-like plate was used to fix the fracture

A 59-year-old female with osteopetrosis presented a history of left hip pain and bilateral, right greater-than-left, knee pain with progressively activity limitation for the past 13 years. She had no previous fracture history. At the time of admission to the Department of Orthopedic Surgery, Wuhan Puai Hospital, the examination revealed a painful left hip and a painful right knee with diminished movement. X ray of the pelvic showed endobones, and x ray of spine showed “rugger jersey spine” (Fig 1). The x-rays also showed severe osteoarthritis in the left hip with significant acetabular erosion, proximal migration of the femoral head and reduced joint space (Fig 1). X-rays of the right knee showed similar osteoarthritic changed (Fig 1). The patient consented for THA in the left hip and TKA in the right knee. A careful planning and preparation was done preoperatively. Total left hip arthroplasty was performed in first instance. The THA operation was performed through a posterolateral approach. The femoral head was with severe degenerative changes, but there were no signs of collapse or avascular caput necrosis. The acetabulum was exposed and prepared before inserting the femoral component (Zimmer, USA). The femoral neck was truncated and the distorted femoral head was removed. After 3 h careful drilling in order to restore the medullary canal in the proximal femur, the greater trochanter accidentally broke off and was fixed with steel-wire (Fig 2). Weight-bearing was not permitted during the first 12 weeks post-operatively, and only partial weight-bearing was allowed for further six weeks. Six months later the patient had recovered from the THA, and a TKA (Depuy, USA) was performed on the right knee. After extra medullary alignment, multiple saws were used to make the femoral and tibial cuts. The cement prosthesis was extremely gently impacted. Nevertheless, an avulsion fracture occurred on the femoral medial epicondyle. A small spider-like plate was used to fix the fracture (Fig 3). The patient was advised to wear knee brace for a week.

**Fig. 4:**
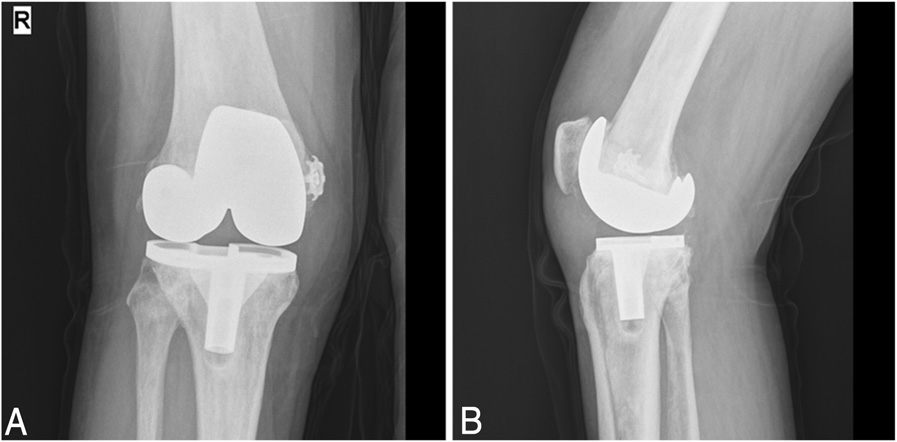
One-year postoperative radiograph of the right knee. Antero-posterior (**a**) and lateral (**b**) views show satisfactory position of the components

At 15-months follow-up, x-rays showed satisfactory position of the components (Fig 4), and the patient could walk freely and perform activities of daily living with no pain.

## Discussion

At the moment no curative treatment is available for benign osteopetrosis. Treatment consists largely of symptomatic management of complications, and is thus supportive. Degenerative osteoarthritis is common in patients with osteopetrosis. Symptomatic joint osteoarthritis in this patient population may be relieved pharmacologically with nonsteroidal anti-inflammatory drugs, but for some cases, total joint arthroplasty may be necessary. A thorough preoperative planning and preparation is of paramount importance for successful THA and TKA in patients with osteopetrosis. The narrow and sometimes missing medullary canal can make intramedullary reaming very challenging, and iatrogenic fractures are likely to occur under this procedure. The sizing of the components may be difficult and cement-bone interdigitation can be challenging due to small surfaces and very dens bone. Finally, these patients are known to be at higher risk of osteomyelitis as a result of poor blood supply to the bone and relative neutropenia.

In our case the greater trochanter was broken off while creating a medullary canal in the extremely hard bone in the proximal femur. It was necessary to fix the fracture with steel-wire. While performing TKA in the right knee an avulsion fracture occurred on the medial epicondyle of the femur. Also this fracture needed to be fixed, and patient was advised to wear knee brace for a week. Bone overheating was also a problem during surgery, and we managed this by careful and low speed drilling while continuous cold saline was administered on the bone. All these intraoperative complications and difficulties increased operation time and thereby the risk of intraoperative infection.

Currently, there are 14 articles in English literature reporting joint arthroplasty in patients with osteopetrosis. These articles (Table 1) consist of 17 THA in 13 patients, 3 hip resurfacing in 2 patients, and 5 TKA in 5 patients. Overall, these procedures have proven to be technically difficult to perform with increased risks of intraoperative and postoperative complications. However, the outcomes have been successful (Table 1).

**Table 1:** The results of total joint arthroplasty in patients with osteopetrosis

Authors	Age	Gender	Surgical treatment	Complications	Follow-up period
Janecki [6]	44	Male	Left cemented THA	Fracture of lesser trochanter	6 months
Cameron [7]	40	Female	Bilateral cemented THA	None	4 years
Casden [8]	50	Female	Left cemented TKA	None	2 years
Ashby [9]	69	Female	Right cemented THA	None	Lost follow-up
Ashby [9]	49	Female	Bilateral cemented THA	Dislocation of one hip	5 years
Matsuno [10]	16	Female	Bilateral hybrid THA	None	6 years
Matsuno [10]	50	Male	Left hybrid THA	None	1 year
Egawa [11]	48	Male	Bilateral hybrid THA	None	10 months
Strickland [12]	45	Female	Left THA	None	3.5 years
Strickland [12]	47	Male	Right THA	None	4 years
Strickland [12]	41	Female	Left THA	A partial sciatic nerve palsy with foot drop	Lost follow-up
Strickland [12]	68	Female	Left TKA	None	20 years
Strickland [12]	42	Female	Right TKA	None	2 years
Girard [13]	34	Female	Left hip resurfacing	none	One year
Ramiah [14]	38	Male	Left THA	None	None
Wang [15]	35	Male	Bilateral metal-on-metal hybrid hip resurfacing	None	25 months
Wang [16]	22	Female	Right THA	None	10 years
Manzi [17]	36	Female	Left THA	None	2 years
Mayer [18]	58	Male	Right TKA	Medial condyle fracture	6 months
Van Hove [19]	41	Female	Right TKA and Right hip resection arthroplasty	Iatrogenic fracture	1 year

Surgical technique differed considerably among the case reports. For THA, Janecki and Nelson [6] described difficulty in making femoral neck cut, difficulty in preparation of femoral canal and minor intraoperative fracture of lesser trochanter. Cameron and Dewar [7] used short femoral stems to minimize the need for reaming of the femoral canal. Egawa et al. [8] reported the use of computer-assisted fluoroscopic navigation to aid in the creation of a femoral canal with a high-speed burr. Girard et al. [9] reported that in order to avoid the difficulties associated with inserting the femoral component, a hybrid metal-on metal resurfacing may be a good option. Ramiah et al. [10] presented technical solutions to aid surgeons in the management of failed proximal femoral internal fixation using custom-made tungsten carbide instrumentation. Our previous article [11] presented a case of arthroplasty for an osteopetrosis patient with 10-year follow-up. At 10-year follow-up, the patient could walk freely and perform activities of daily living with no pain. For TKA, Mayer [12] presents a case of knee osteoarthritis in a patient with osteopetrosis. Patient-specific instrumentation was used in this case. This technique decreases operative time and potential complications. Van Hove [13] reported that TKA in autosomal dominant type I osteopetrosis was related with iatrogenic fractures. Total hip arthroplasty was performed during follow-up, but there was no possibility of achieving a stable THA. Therefore, they performed a resection arthroplasty. The patient was able to walk a few steps behind a walking aid.

## Conclusions

Our case report demonstrates that total joint arthroplasty is an effective option to treat hip and knee osteoarthritis in patients with osteopetrosis. This case highlights the technical difficulties and complications associated with total joint arthroplasty in osteopetrosis patients. Operative time is increased significantly secondary to hard sclerotic bone, obliterated medullary canal and perioperative fractures. Careful preparation, appropriate instruments and knowledge is necessary to achieve successful outcomes.

## Consent

Written informed consent was obtained from the patient for publication of this case report and any accompanying images. A copy of the written consent is available for review by the Editor-in-chief of this journal.

## Abbreviations

THA: Total hip arthroplasty
TKA: Total knee arthroplasty
